# Supplementary material for: Measuring multiple evolution mechanisms of complex networks
Source: Sci Rep. 2015 Jun 11;5:10350. doi: 10.1038/srep10350 (PMC4464182; doi:10.1038/srep10350)
Supplement: Supporting Information [file srep10350-s1.pdf]

Measuring multiple evolution mechanisms of complex networks

Qian-Ming Zhang, Xiao-Ke Xu‡, Yu-Xiao Zhu, Tao Zhou§

Corresponding to: ‡ xiaokeeie@gmail.com, § zhutou@ustc.edu

Supplementary Information

Estimation through Improved Link Prediction Index

In the main text, we claimed one disadvantage of the Link Prediction method is due to its rough descriptions of links. So we redefined the Common neighbor (CN) index to keep in step with the likelihood analysis method. However, the estimation is still incorrect as shown in Figure S1.

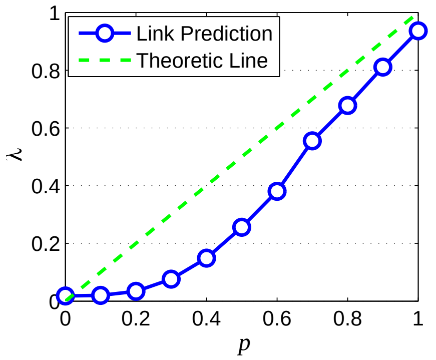

Figure S1. Correlation between the optimal λ and p. p is the known proportion of clustering mechanism compared to popularity mechanism. λ is the estimated value by the improved link prediction method.

Estimating the minimum number of n in Calculating AUC

The calculation of AUC is very similar to a Bernoulli experiment, where there are only two results — success or failure and every result is independent to each other. Suppose the exact value of AUC is q, we can get score 1 with probability q and score 0 with probability $1-q$ when we sample node pairs to calculate AUC. Among n independent Bernoulli experiments, if we get n' positive results, n'/n will be the approximated AUC value. Obviously, the larger n is, the better the approximation is. Then we can estimate the minimum effective value of n, denoted as n^*, which is able to ensure the standard deviation of AUC is small enough. In the n independent Bernoulli experiments, if we record positive result as A,

$q = p(A)$, we can get $P_n(k) = C_n^k q^k (1-q)^{n-k}$, where $k = 0, 1, ...n$; $P_n(k)$ means the probability we get k positive results out of n experiments.

We can treat the n samples as the random variables X_i, $i = 1, 2, ..., n$, where X_i obeys Bernoulli distribution $\mathbf{B}(1, q)$. Then we can get its expectation $E(X_i) = q$, and variance $D(X_i) = q(1-q)$. According to the Central Limit Theorem, we can get

$$Y_n = \frac{1}{n}\sum_{i=1}^{n} X_i \quad \sim \quad N(q, \frac{q(1-q)}{n}),$$

where Y_n represents the frequency of the positive result A occurs. The n^* can be defined as the minimum number that makes $Y_n - q$ as small as enough, namely $P(|Y_n - q| < \varepsilon) > C$, where ε is a small number indicating the margin of error and C is the confidence level. Then this equation can be rewritten as $\Phi(\frac{\varepsilon}{\sqrt{q(1-q)/n}}) - \Phi(\frac{-\varepsilon}{\sqrt{q(1-q)/n}}) > C$, where $\Phi(\cdot)$ is the integral function of the normal distribution. As $\Phi(-s) = 1 - \Phi(s)$, we can get $2\Phi(\frac{\varepsilon}{\sqrt{q(1-q)/n}}) - 1 > C \Rightarrow \Phi(\frac{\varepsilon}{\sqrt{q(1-q)/n}}) > \frac{C+1}{2}$. Considering $\varepsilon = 0.001$ and $C = 90\%$, we have $\Phi(0.001\sqrt{\frac{n}{q(1-q)}}) > 0.95$. By checking the normal distribution table, we know $\Phi(1.64) = 0.9495$. So we can easily obtain $\sqrt{n} > 1640\sqrt{q(1-q)}$. As a result, if $q > 0.5$, namely AUC > 0.5, n^* will be smaller and smaller as q grows. For any given $q > 0$, $0 < q(1-q) \leq 0.25$, we can get $n > 674200$. This is the upper bound of n^*. Thus we can conclude that if we expect to get the AUC value with error less than 0.001 at the 90% confidence level, 672400 will be large enough for n.
